# Improving detection of mental health problems in community settings in Nepal: development and pilot testing of the community informant detection tool

**DOI:** 10.1186/s13031-017-0132-y

**Published:** 2017-11-20

**Authors:** Prasansa Subba, Nagendra P. Luitel, Brandon A. Kohrt, Mark J. D. Jordans

**Affiliations:** 1 Transcultural Psychosocial Organization (TPO) Nepal, Kathmandu, Nepal; 2 The Department of Psychiatry and Behavioral Sciences, George Washington University, Washington, DC 20037 USA; 3 Department of Research and Development, HealthNet TPO, Amsterdam, The Netherlands; 4 Center for Global Mental Health, King’s College London, London, UK

**Keywords:** Detection, Mental health, Treatment, Community informant, Help-seeking, Low- and middle-income countries, Nepal

## Abstract

**Background:**

Despite increasing efforts to expand availability of mental health services throughout the world, there continues to be limited utilization of these services by persons with mental illness and their families. Community-based detection that facilitates identification and referral of people with mental health problems has been advocated as an effective strategy to increase help-seeking and service utilization. The Community Informant Detection Tool (CIDT) was developed for the community informants to identify people with depression, psychosis, alcohol use problems, epilepsy, and child behavioral problems in community settings. The CIDT has been validated in Nepal and found to be effective in promoting treatment initiation. To facilitate replication in other settings, this paper describes the development process of CIDT and the steps to achieve comprehensibility, utility and feasibility.

**Methods:**

The CIDT was developed in four steps. First, case vignettes and illustrations were created incorporating local idioms of distress for symptoms of each disorder with an expert panel of 25 Nepali mental health professionals. Second, the utility of a draft tool was assessed through focus group discussions (*n* = 19) and in-depth interviews (*n* = 6). Third, a practice run was conducted assessing applicability of the tool through IDI among purposively selected community informants (*n* = 8). Finally, surveys were administered to 105 community informants to assess feasibility.

**Results:**

The first through third steps led to modifications in the format and presentation of the CIDT. The pilot test found CIDT to be comprehensible and feasible for detection and referral of all conditions except child behavioral problems. Female community health volunteers were recommended as the most appropriate persons to utilize the CIDT.

**Conclusion:**

Community-based detection using the CIDT for persons in need of mental health care is perceived to be useful and feasible by key community stakeholders who would integrate the tool into their daily activities.

**Electronic supplementary material:**

The online version of this article (10.1186/s13031-017-0132-y) contains supplementary material, which is available to authorized users.

## Background

Challenges in reducing the global burden of disease attributable to neuropsychiatric disorders can be categorized into two groups: supply-side challenges and demand-side challenges. Jacobs et al. [1] define supply-side challenges as health system constraints hindering service uptake at the individual, household and community level and demand-side challenges as factors impeding individual, household, and communities’ timely use of health services. In this article, we focus on demand-side challenges and barriers to accessibility and availability of mental health services at the community level. Supply-side challenges refer to the lack of mental health services, especially in low-resource settings emblematic of low- and middle-income countries (LMIC) [2]. For example, there is an estimated gap of 1.18 million providers of mental health services in LMICs [3]. Supply-side challenges are being addressed predominantly through task-shifting, also known as task-sharing, models in which non-specialists including primary care and community health workers are trained to take on responsibilities typically in the purview of mental health specialists such as psychiatrists, psychologists, and clinical social workers [4, 5]. The World Health Organization mental health Gap Action Programme (mhGAP), which includes an intervention guide and training curricula on mental health for primary care workers, is a keystone approach to this endeavor worldwide [6]. Adopting this approach, the Programme for Improving Mental Health Care (PRIME) has mitigated supply-side challenges in Nepal through the integration of mental health services in the primary health care centres [7].

Unfortunately, as the supply-side issues begin to be addressed, there continue to be demand-side challenges in which persons with mental illness and their families do not seek mental health services [2, 7, 8]. Stigma associated with mental illness, lack of recognition of mental health problems and lack of awareness regarding where to go for treatment contribute to this under-utilization [8, 9]. In high-resource settings, where populations regularly interact with healthcare providers, detection of mental disorders is commonly based on assessments by trained personnel at health care facilities using structured screening tools [10]. However, in settings with limited health infrastructure and diverse cultural attitudes regarding the etiology of and appropriate ways of seeking help for mental illness, health facility-based screening may be inadequate to detect persons needing care. Moreover, in LMIC there is often a long delay in initiating treatment-seeking. One study in Nepal found that none of the women screening positive for maternal depression had sought care, even when the required service was available in local primary health facilities [11]. Another study found that the duration of untreated psychosis can average 2 years, and it is not uncommon to go more than 5 years without initiating treatment [12]. Delayed biomedical treatment-seeking may reflect a tendency to seek traditional healing services first as well as lack of experience identifying symptoms of mental health problems and lack of knowledge about available services for treatment. Therefore, reducing the delay in treatment-seeking is an important public health goal related to demand-side barriers.

An alternative approach proposed to increase access to care in LMIC is community case-finding [13]. This is done by identifying a case at the community level and then completing a referral to a health facility where a diagnostic interview by a trained health worker confirms diagnostic status and initiates treatment [14]. Community case finding by lay community workers, particularly in resource-poor settings, could be advantageous in several ways: community health workers have wide coverage [15]; they can mobilize patients to engage in services at health facilities; they are a sustainable resource with a strong knowledge of the local population’s health needs [16]; and they have higher acceptance from patients and their families than mental health specialists [17]. In Nigeria and India, a case-finding effort conducted by the community workers was found to be effective in identifying people with mental health problems [18].

To facilitate mental health problem identification at the community level in Nepal, we developed the Community Informant Detection Tool (CIDT) [19], an innovative and culturally appropriate tool for detection and referral of persons with one of four target mental health problems included in PRIME: depression, alcohol use problem, psychosis, and epilepsy. As child mental health problems are frequently neglected and children are rarely brought for care [20, 21], we added child behavioral problems to explore the potential for engaging children in services. The tool comprises of case vignettes for each mental health problem using local non-stigmatizing idioms accompanied by illustrations. Community informants are asked to match and specify the degree of matching of people in their surroundings with the prototype vignettes, determine the general impact on daily functioning, and assess willingness to seek care. The probable positive cases identified through the tool are encouraged to visit the health facility, where a clinical assessment is done by trained primary care health workers.

A recent validation study shows that this process of identifying and referring cases is effective for increasing utilization of mental health care in a Nepali context. The study found that 64% of the people identified as probable cases by community informants using the CIDT were actually diagnosed with a mental health problem in the clinical interviews [19]. Another study evaluating the CIDT highlights that two-thirds of the total cases identified by community informants using CIDT actually sought care and 77% of those who sought care initiated treatment at the health facility within 3 weeks [22]. These studies indicate that CIDT is appropriate and advantageous to use in resource-poor, rural communities and can be scaled up in similar contexts when culturally adapted. Researchers seeking to scale up the tool can follow the stepwise development process presented in this paper and adapt CIDT in their context. This paper also describes findings from the formative research which assessed the tool in three aspects: (a) comprehensibility- easily understood by people with basic literacy and is user friendly; (b) utility: usefulness of the tool to identify probable cases of mental health problems; and (c) feasibility: possibility to integrate the tool in routine work and to convince community members to seek care.

## Methods

### Setting

This study was conducted in Chitwan, a district in southern Nepal. Nepal is one of the poorest countries in the world. The country suffered a decade long conflict between the state government and the Maoists from 1996 to 2016 claiming more than 13,000 lives. The recent earthquakes in April and May 2015 claimed nearly 9000 lives and unleashed mental health problems in many. Previous studies have shown evidence of increased mental health problems in the earthquake affected population [23, 24]. While the mental health needs are large, there are limited resources. It is estimated that there are 0.22 psychiatrists and 0.06 psychologists per 100,000 persons in the country [25]. To address this lack of services, PRIME was initiated in Nepal, with a pilot primary care-based mental health integration program in Chitwan. Although specialized mental health services from psychiatrists were available in Chitwan through tertiary level health facilities, mental health services were non-existent in the community-based health facilities prior to PRIME implementation [9].

### Ethical approval

This study was conducted as a part of a larger study PRIME. The study protocol was reviewed and approved by the Nepal Health Research Council. Informed consent form explaining about the study, its procedure, benefit and confidentiality of the data was administered and a written informed consent was obtained from all the participants prior to data collection.

### Data collection and analysis

Data collection was conducted by 4 researchers having at least an undergraduate degree. All researchers had previous working experience in mental health and research. They attended an additional 2-week training on the nature of the study and research instruments. The data collection took place in the community at the respondents’ residences. Each qualitative interview lasted about 30–45 min and was digitally recorded. The interviews were transcribed, and then translated into English by a professional translator. The interviews were qualitatively analyzed using the thematic approach based on a priori themes in NVivo10 [26] to explore: (i) informants’ perceptions of the tool; (ii) comprehensibility; (iii) utility; (iv) feasibility of the tool and; (v) suggestions and recommendations to make the tool more context-appropriate. Survey data were coded and entered into SPSS16 for analysis [27]. Frequency table, cross tabulation, and mean value were generated. Further analysis was done to find mean difference between groups using one-way ANOVA. Significance (*p* < 0.05) for mean difference between each group was calculated using Tukey post-hoc test.

### Study procedure

We conducted a four-step process to develop the CIDT and evaluate its comprehensibility, utility, and feasibility.

#### Step 1: Development of draft tool

First, to develop a case vignette, a list of symptoms for each of the five problems was prepared following the WHO mhGAP intervention guide (mhGAP-IG) [6] and prior ethno-psychological research [28]. Second, prioritization of relevant symptoms to be included in the case-specific vignette was done by an expert panel of 25 Nepali mental health professionals (17 psychiatrists, 6 psychologists, 2 psychosocial counselors). The expert panel was asked to rate relevance of symptoms on a three-point scale [3 = “high relevance” - everyone, including the uneducated, can understand the term, and the symptoms are common among the people with this problem; 2 = “low relevance” - most people with at least a primary education level can understand the term, and/or the symptoms are seen in most people with this problem; and 1 = “not relevant” - only health workers or people with advanced education can understand the term and/or symptoms are rarely seen in people with this problem]. Third, a case vignette integrating the prioritized symptoms in local terms/idioms were developed for each of the five problems. Three structured questions were included at the end of each case vignette: a) Does this narrative apply to the person you are talking to now?; b) Does this person want support in dealing with these problems?; and c) Do the problems have a negative impact on daily functioning? The community informant asks these questions to assess whether a person needs referral to the local primary care facility. The response to the first question was on a sliding scale (“no match,” “moderate match,” “good match,” and “very good match”) and the other two questions had a “Yes/No” response format. Potential caseness was then defined as matching at the “moderate” level or above and having recognizable impairment in daily life and/or desire to seek support.

#### Step 2: Qualitative study

To assess the level of understanding of the vignettes amongst community informants, a total of two focus group discussions (FGDs) (one with 9 Female Community Health Volunteers [FCHV] and one with 10 members of mothers’ groups) and six in-depth interviews (IDIs) (2 each with traditional healers and pharmacists and 1 each with a member of a mothers’ group and a FCHV) were carried out with participants coming from two village development committees (VDC) where the PRIME Mental Health Care Package (MHCP) was pilot tested. The selection of community informants was based on the results from prior qualitative formative research [2] that identified “trusted” and “respected” community figures, who could play a role in detection and referral of people with mental health problems in the community. We selected FCHVs, traditional healers, pharmacists, and members of mothers’ groups for this study.

#### Step 3: Practice run

To assess feasibility of using the tool in daily life, we selected eight community informants (two from each group: FCHVs, mothers’ group members, traditional healers, and pharmacists). These community informants were engaged in a half-day training on the basic concepts of psychosocial and mental health problems, the CIDT, its administration procedure and ethical considerations. They were then asked to use the tool within their daily routine. In order to demonstrate respect for the existing role of traditional healers in the management of mental health problems, we encouraged the traditional healers to continue their local practices but refer only the suspected mental health problems which they would not be able to treat. One week after the training, the community informants were asked about their experiences using the tool in their daily lives, with probing questions regarding improvements to be made in the tool.

#### Step 4: Survey

A survey of 105 community informants (FCHVs- 25, member of mothers’ group- 27, traditional healers- 26, and pharmacists- 27) was conducted in which each participant was given one of the five CIDT tools. The vignettes were first read out to the participant, followed by structured questions about the tool. The questions addressed comprehensibility, willingness to use the tool, ability of the tool to detect people and promote help seeking, and appropriate cadres of community members to use the tool. Each question was rated on a 4-point scale where 1 meant not at all and 4 meant very much. Of the total 105, 22 (21%) of the respondents were asked questions on the depression vignette, 19 (18%) on psychosis, 20 (19%) on alcohol use problems, 22 (21%) on epilepsy, and 22 (21%) on child behavioral problems.

## Results

### Step 1: Development of the draft tool

Altogether, 26 symptoms were listed out for depression, 18 for psychosis, 17 for alcohol use problem, and 19 for child behavioral problems. For epilepsy, the listed key symptoms in mhGAP were translated to Nepali. At least 8–10 symptoms rated as highly relevant by the experts (See Table 1) were incorporated in each vignette (See Vignette Example) followed by two pictures illustrating “perceived need for support” and “difficulties in daily functioning”.

**Table 1:** Top five most relevant symptoms per condition as rated by mental health experts (*N* = 25)

Depression		
Symptom (English)	Symptom (Nepali idioms)	Relevance rating, Mean (SD)
1. Depressed mood	To become depressed or sad for a very long time; *“udaas wa niraas hunu,” “lamo samayasamma/nirantar roopma dukhi hunu,” “man* ^a^ *-dukhnu,”*- *“man bhari hunu,” “man khinna hunu,” “birakta lagnu”*	2.84 (0.47)
2. May attempt suicide	Attempts of suicide or self-harm; *“aatma hatya garna khojne,” “afailai haani honey kriyakalap garne”*	2.71 (0.56)
3. Disturbed sleep	Not being able to sleep at night or restlessness; *“nindra nalagne,” “nindrama gadbadi,” “nidauna garho huney”*	2.70 (0.637)
4. Life is not worth living	Sees no point or meaning in living, feels that life is worthless; *“baachna man nalagne,” “jiwan bekaar lagne,” “bachnako kunai saar/artha chaina jasto lagne”*	2.74 (0.44)
5. Anger and irritability	Easily angered or irritated or frustrated over trivial matters; *“sa-sana kurama ris uthne,” “jharko lagne,” “dikka lagne”*	2.68 (0.56)
Alcohol use problems		
1. Slurred speech	Slurred/unclear speech; *“boli larbaraune,” “boli bujhna nasakne,” “prasta boli nahune”*	2.86 (0.35)
2. Uninhibited behavior	Acts or speaks on their own will, unashamed; *“man lagyo tyehi garne/bolne,” “laaj namanne”*	2.86 (0.35)
3. Drinks alcohol frequently	Drinks too often; *“barambaar jaad-rakshi piune”*	2.82 (0.39)
4. Difficulties in controlling alcohol use	Not able to stop or control once drinking starts, strong urge to drink alcohol all the time; *“jaad-rakshi khana thalepachi rokna nasakne wa afailai niyantran garna nasakne,” “jaad-rakshi matra khairahana man lagne/khairahane”*	2.78 (0.51)
5. Smell of alcohol on breath	Strong smell of alcohol on breath most of the time; *“sadhai jaso jaad-rakshi ko gandha aaune”*	2.76 (0.52)
Psychosis		
1. Incoherent or irrelevant speech	Others could not comprehend what they say, irrelevant speech, says whatever they like; *“boleko kura aruharule bujhna nasakne,” “aasandharbhik kura bolne wa j payo tyehi bolne”*	2.92 (0.40)
2. Withdrawal, agitation, disorganized behavior	Withdrawal, prefers to stay alone, shows irrelevant or disorganized behavior or activities; *“chutiyera basne/eklai basna ruchaune,” “manma ashanti huney,” “asandharbhik/aabewasthit bewahar tatha kriyakalap garne”*	2.84 (0.37)
3. Difficult to stop or talk to	Talks too much and difficult to stop; *“boleko bolekai garne,” “bolna bata rokna garho huney”*	2.76 (0.43)
4. Social withdrawal and neglect of usual activities	Social withdrawal, neglect of usual activities (such as work, school, neglect of family or social responsibilities); *“samajik kriyakalapharu bata alaggiyera basne,” “dainik kriyakalapharu prati bewastha garne”*	2.70 (0.47)
5. Hallucinations	Hears sound that others cannot hear; *“waripari kehi nahuda pani kohi boleko wa kehi awaaz sunne”*	2.68 (0.55)
Child behavioral problem		
1. Frequent and severe temper tantrums	Easily angered, loses temper over trivial things; *“chado sa-sana kurama dherai jhokkiney, risaune”*	2.81 (0.40)
2. Excessive talking or noisiness	Talks often, very noisy, can’t sit without talking; *“dherai bolne,” “dherai halla garne,” “naboli basna nasakne”*	2.76 (0.43)
3. Running away from home	Running away from home; *“ghar bata bhagne”*	2.71 (0.46)
4. Over-activity (excessive for the context or situation)	Too mobile, restlessness or cannot stay in one place; *“ekdumai chali rahane wa daudirahane,” “chaatpatayeko jasto huney,” “shaanta bhayera basna nasakne,” “dherai chuk-chuk garne”*	2.70 (0.55)
5. Truancy from school	Absenteeism/does not attend school regularly; *“bidhalaya anupasthit huney, aniyamit roopma janey”*	2.68 (0.64)

^a^ *man-*See Kohrt, B. A., & Harper, I. (2008). Navigating diagnoses: Understanding mind–body relations, mental health, and stigma in Nepal. *Culture, Medicine, and Psychiatry*, *32*(4), 462

**Table:** 

**Vignette Example: Case vignette of alcohol use problem**
Rajan drinks alcohol all the time, due to which, whenever someone goes near him, one can smell the strong stench of alcohol emanating from him. Because he always drinks alcohol, his speech is slurred and others find it very difficult to understand him. As he craves alcohol every day, he keeps consuming alcohol. After drinking alcohol, he speaks or does whatever he likes. Once he starts drinking alcohol, he cannot control himself and he always ends up drinking a lot. Due to heavy drinking, he has trembling limbs, sweats profusely, feels restless, and has increased palpitations. These days he no longer finds pleasure in activities he used to enjoy earlier, instead he has started to become engrossed in drinking alcohol. Due to such behavior, he is not able to complete his daily activities.

### Step 2: Qualitative study

The qualitative study participants found the vignettes comprehensible as they used simple words and presented common symptoms in local idioms. Although most of them found using the vignettes to identify potential cases feasible, a few described having difficulty as they had limited knowledge on mental health. To address this, they recommended conducting training prior to the tool’s use and using large, clear and colorful pictures for the vignettes. Further, they preferred the “yes/no” option for “daily functioning impairment” and “need for support” while preferring multiple response options for the decision about the case referral. As one participant explained,

*“[....] if I say “yes”, it will be something like giving a decision. It is not wise for us to identify mental health problems only based on the symptoms mentioned in the vignette. -IDI with pharmacist.”*

### Step 3: Practice run

Based on the findings from the qualitative study, multiple response options were kept for the first question about degree of match whereas the dichotomous yes/no response options were kept for the second and third questions. Two colorful pictures presenting a sad woman sitting passively and leaving work undone and a man supporting another man to visit the health post were included in the second and third questions to illustrate functional impairment and need for support. A half-day training was organized with 8 community informants prior to the practice run.

After a week of integrating the tool in their daily lives, the community informants found the case vignettes of psychosis, alcohol use problem and epilepsy easy to understand and useful to identify potential cases. Case vignettes of depression and child behavioral problems were the most difficult to use, for which they recommended intensive training, incorporating associated pictures for symptoms and organizing community awareness programs. Concerning referral, the community informants were comfortable referring cases with “high match” with the vignette. The three common methods used by the community informants to identify potential mental health cases were: (a) observation of the behavior of suspected case, match and refer; (b) read out the vignettes to the community and fill out the CIDT form with their help; and (c) consulting the family members of the suspected person, fill out the form and motivate to visit the nearby health facility. Although home visit was endorsed as the best method to proactively identify mental health cases, all except the FCHVs found it impractical.

Overall, the community informants were willing to use the tool in their daily lives. They took it as *“an opportunity to help people in need”* and experienced a sense of *“gratification”* when doing so. Being able to identify and refer potential cases was also perceived as a part of *“capacity building*.*”* Although everyone was willing, not all community informants found it feasible to engage in the task fully. They rather nominated FCHVs and members of mothers’ groups as the best people to take up the task since they have a strong network in the community. Upon carrying out this task, the importance of providing incentives, preferably in a monetary form, was underscored by all the informants.

### Step 4: Survey

A survey was conducted with 105 community informants, where most of them were female (66.7%), Brahman/Chhetri (63.8%), Hindu (93.3%), between 16 and 75 years old (*M* = 43.62, *SD* = 12.36), and had a minimum of a secondary level of education (53.3%), with the exception of traditional healers, none of whom had completed secondary education (Table 2).

**Table 2:** Socio-demographic characteristic of the respondents

	FCHV (*n* = 25)	Mothers’ group (*n* = 27)	Traditional Healers (*n* = 26)	Pharmacists (*n* = 27)	Total (*N* = 105)
Sex					
Male	–	–	22 (84.6%)	13 (48.1%)	35 (33.3%)
Female	25 (100%)	27 (100%)	4 (15.4%)	14 (51.9%)	70 (66.7%)
Caste/Ethnicity					
Brahman/Chhetri	19 (76%)	17 (63%)	12 (46.2%)	19 (70.4%)	67 (63.8%)
Tharu	4 (16%)	2 (7.4%)	5 (19.2%)	2 (7.4%)	13 (12.4%)
Ethnic Groups	1 (4%)	5 (18.5%)	5 (19.2%)	5 (18.5%)	16 (15.2%)
Others	1 (4%)	3 (11.1%)	4 (15.4%)	1 (3.7%)	9 (8.6%)
Religion					
Hindu	25 (100%)	25 (92.6%)	22 (84.6%)	26 (96.3%)	98 (93.3%)
Buddhist	–	1 (3.7%)	4 (15.4%)	1 (3.7%)	6 (5.7%)
Christian	–	1 (3.7%)	–	–	1 (1.0%)
Age groups					
Up to 24	–	1 (3.7%)	1(3.8%)	2 (7.4%)	4 (3.8%)
25–59	25 (100%)	26 (96.3%)	13 (50%)	25 (92.6%)	89 (84.8%)
60+	–	–	12 (46.2%)	–	12 (11.4%)
Education					
Illiterate	–	2 (7.4%)	9 (34.6%)	–	11 (10.5%)
Non-formal education	3 (12.0%)	6 (22.2%)	7 (26.9%)	–	16 (15.2%)
Primary and secondary level	22 (88%)	16 (59.3%)	10 (38.5%)	8 (29.6%)	56 (53.3%)
Higher Secondary or above	–	3 (11.1%)	–	19 (70.4%)	22 (21%)

#### Perception of the tool by type of respondent

**Fig. 1:**
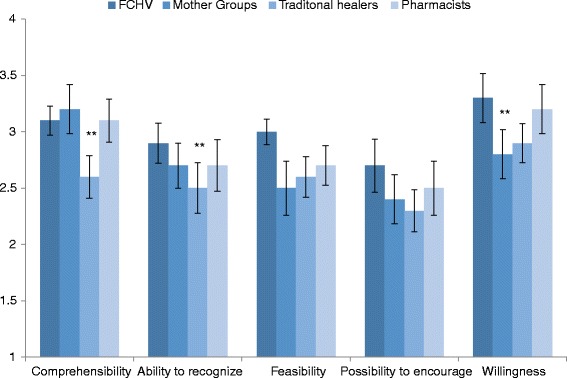
Perception of the tool by the type of respondents (*N* = 105)

The tool was perceived to be highly comprehensible (M = 3.02, S.D. = 0.52), moderately feasible to use to recognize the targeted problems (M = 2.72, S.D. = 0.56), useful in daily life (M = 2.74, S.D. = 0.50) and able to encourage those positively identified to seek care (M = 2.53, S.D. = 5.8). The community informants were fairly willing to take up the task (M = 2.72, S.D. = 0.56). There were statistically significant differences in mean scores for comprehensibility of the vignettes (*p* = <0.01), ability to recognize problems (*p* = 0.03) and willingness to use the vignettes (*p* = 0.01) between different groups of community informants as determined by one-way ANOVA. The comprehensibility ratings of the traditional healers were significantly lower compared to those of the mothers’ group members (*mean diff.* = 0.56; *p* < 0.01) and FCHVs (*mean diff.* = 0.46; *p* = 0.05). Similarly, the traditional healers’ ability to recognize potential cases was significantly lower than that of FCHVs (*mean diff.* = 0.46; *p* = 0.01) and the mothers’ group members’ willingness to use the tool was significantly lower than that of FCHVs (*mean diff.* = 0.43; *p* = 0.02). (See Fig. 1).

#### Community informants’ perception of the vignettes by type of problem

Overall, the community informants indicated moderate to high levels of comprehensibility of the vignettes (M = 3.02, SD = 0.52) and they were moderately to highly willing to take up the task of using the tool in their daily lives (M = 3.09, SD = 0.56). The case vignette for child behavioral problems was least comprehensible (M = 2.86, SD = 0 .46) and the community informants were least confident in their ability to recognize potential cases through this vignette (M = 2.27, SD = 0.55). Comparatively, they were quite hesitant to motivate individuals to seek care in cases of alcohol use problem (M = 2.3, SD = 0.57) and child behavioral problems (M = 2.3, SD = 0.47) (Table 3).

**Table 3:** Community informants’ perceptions of the vignettes by type of problem

Type of problem	Comprehensibility	Ability to recognize	Feasibility to use in daily life	Possibility to encourage to seek care	Willingness to take up the task
	Mean (SD)	Mean (SD)	Mean (SD)	Mean (SD)	Mean (SD)
Depression (*n* = 22)	3.18 (0.66)	2.77 (0.68)	2.81 (0.50)	2.72 (0.70)	3.18 (0.73)
Psychosis (*n* = 19)	3.00 (0.47)	2.78 (0.41)	2.78 (0.53)	2.73 (0.56)	3.15 (0.50)
Alcohol use problem (*n* = 20)	3.00 (0.56)	2.90 (0.55)	2.80 (0.52)	2.30 (0.57)	3.05 (0.51)
Child behavioral problems (*n* = 22)	2.86 (0.46)	2.27 (0.55)	2.54 (0.50)	2.31 (0.47)	3.04 (0.57)
Epilepsy (*n* = 22)	3.09 (0.42)	2.90 (0.29)	2.77 (0.42)	2.59 (0.50)	3.04 (0.48)
Total (*N* = 105)	3.02 (0.52)	2.72 (0.56)	2.74 (0.50)	2.53 (0.58)	3.09 (0.56)

#### Right person to take up the task

The community informants recommended FCHVs (47.6%) and mothers’ groups (23.8%) as suitable cadres for using CIDT (See Table 4). Despite being frequently nominated, the FCHVs perceived themselves as the least fit for the task. They rather recommended mothers’ groups (70.4%) and pharmacists (59.3%). The traditional healers were the only group who perceived themselves as the most appropriate for this task. Contrastingly, none of the pharmacists saw themselves as the right group.

**Table 4:** Perception on the right person to use CIDT

	FCHV (*n* = 25)	Mothers’ group (*n* = 27)	Traditional Healers (*n* = 26)	Pharmacists (*n* = 27)	Total (*N* = 105)
FCHV	7 (28.00%)	19 (70.40%)	8 (30.80%)	16 (59.30%)	50 (47.60%)
Mothers’ group	12 (48.00%)	7 (25.90%)	1 (3.80%)	5 (18.50%)	25 (23.80%)
Traditional healer	2 (8.00%)	–	8 (30.80%)	1 (3.70%)	11 (10.50%)
Others^a^	4 (16.00%)	–	2 (7.60%)	4 (14.80%)	10 (9.80%)
Don’t know	–	1 (3.70%)	7 (26.90%)	1 (3.70%)	9 (8.60%)

^a^Includes teachers, social workers/mobilizers, health workers/pharmacists, club members, etc

#### Incentives

More than half (60%) of the community informants were in favor of incentives whereas slightly fewer (28.6%) were against it and the remaining (11.4%) were neutral. More FCHVs (80%) were in favor of incentives compared to other respondents (See Table 5).

**Table 5:** Perception on the need of incentives for community volunteers to use these vignettes

Need of incentives	FCHV (*n* = 25)	Mother’s group (*n* = 27)	Traditional Healers (*n* = 26)	Pharmacists (*n* = 27)	Total (*N* = 105)
Yes	20 (80.00%)	16 (59.30%)	12 (46.20%)	15 (55.60%)	63 (60.00%)
No	3 (12.00%)	11 (40.70%)	6 (23.10%)	10 (37.00%)	30 (28.60%)
Neutral	2 (8.00%)	–	8 (30.80%)	2 (7.40%)	12 (11.40%)

### Recommendations

More than half (53.3%) of the survey participants did not have any comments or suggestions. Those who did recommended including large, colorful and clear pictures for all symptoms and of both genders in all the vignettes. Adding symptoms and translating the vignettes into a local ethnic language were also recommended by a few.

## Discussion

The Community Informant Detection Tool (CIDT) was developed with the aim of promoting help-seeking behavior of people with mental health problems through pro-active case finding in the community. We followed several steps including prioritization of the symptoms of five mental health problems based on the relevance of each symptom in the Nepali context; development of vignettes from the findings; a practice run followed by a qualitative study to explore feasibility and applicability using the tool in a real-life setting; and a survey to pilot test the tool. The tool consists of a vignette presenting common symptoms of each targeted mental health problem, where a community informant matches the symptoms of an individual with the prototype on a 5-point scale. A similar approach of “prototype diagnosis” has been found advantageous in clinical settings in the United States [29] but the tool described here is, to the best of our knowledge, the first of its kind to be used in a community setting in LMIC [19]. The use of local terms and idioms was further perceived to be easily comprehensible even by those with limited education. For easy usage in the community setting, the final layout of the tool was modified, colorful pictures depicting symptoms were added and yes/no response options were used for the additional two questions about “daily functioning impairment” and “need for support.” See “Additional file 1” for the finalized tool. This tool is deemed to facilitate detection at the community level followed by referral to a health facility where clinical assessment can be carried out.

In a resource-poor country like Nepal where mental health professionals are scarce, use of checklists for the purpose of screening may not be feasible since it requires mental health expertise [30]. This approach may further be problematic in LMICs because such checklists are usually developed in high-resource settings and lack sensitivity to local contexts. Kleinman [31] discusses that expression, experience and treatment of illness varies across cultures, emphasizing that these cultural attributes should not be ignored in psychiatry. A culturally sensitive intervention is effective in a manner such that it improves acceptance, detection and treatment adherence [5, 32]. We believe the CIDT provides an innovative and context-sensitive approach to case detection which accounts for local culture through the development of simple contextualized vignettes. Our study findings reconfirm that anyone with minimum literacy can use the tool to identify and refer potential mental health cases for treatment [19, 22]. However, there was some preference amongst the community informants regarding the right group to use the tool.

FCHVs and members of mothers’ groups were endorsed as the most appropriate people for using the CIDT. This may be because they are generally from the same community they work in and can mobilize people more effectively. FCHVs are the health volunteers mobilized by the Nepal government to tackle low utilization of services by increasing demand and access to care at the community level [33, 34] and members of mothers’ groups are oftentimes highly engaged in community development and social reform activities. Because of their involvement in community level activities, both groups have good networks and are also respected in their communities. Although FCHVs suggested mothers’ groups as the most appropriate for this task, mobilizing FCHVs to use the CIDT has several benefits. Because FCHVs are already a part of the government system, mobilizing them is more sustainable [33]. Further, they already carry out many health-related activities, hence they can integrate CIDT in their routine work. Unlike mothers’ group members, most FCHVs are literate and therefore can administer the tool more easily. Despite these advantages, only 28% of FCHVs in our study saw themselves as the appropriate group for this task. This could be because they are already overburdened with work and, since they are mobilized by the government as volunteers, they only receive a nominal amount as compensation. As indicated by our community informants, for motivation and quality of work, they need to be remunerated. Failure to pay them well may severely impact the program. It is noteworthy that a similar program in Uganda had to stop mobilizing community volunteers because of budget constraints [16]. Remuneration can be provided monetarily as a flat rate rather than on the basis of case referrals, which might pose risk of over-detection and over-referral.

While CIDT as a tool is comprehensible and feasible, the case vignettes for depression and behavioral problems were perceived as the most difficult ones for case identification. In the case of depression, this could be because (a) its symptoms are more related to mood and emotion, and its experiences are more subjective [35], (b) its symptoms are mostly expressed in terms of psychosomatic complaints [36, 37] and (c) it is often comorbid with other chronic illnesses [38–41]. Contrastingly, our other study findings showed that the community informants more accurately detected depression than any of the other mental health problems when they were given a 1 day training, comprised of both theoretical and practical sessions [19]. In the case of behavioral problems, our community informants considered child behaviors such as disobedience, hyperactiveness, school truancy, picking fights with friends or family, and vandalizing things as normal for that age group. Many were unsure if such behaviors should be labeled as mental health problems requiring support from a mental health professional. Behavioral problems were understood more in terms of illicit drug use or substance use, with only children displaying these behaviors deemed in need of support, which is consistent with the findings of another study conducted particularly on behavioral problem in Nepal [20]. Therefore, a right story that is culturally relevant to the common community beliefs combined with clearer pictures for each subsequent symptom as suggested by the study findings could be a viable option. Also, community informants may not be effective at all times for the identification of all types of mental health problems. Eliciting information from the parents or other immediate contacts involved in the child’s care can be more advantageous in cases of behavioral problems since they can provide accurate and detailed information [42–44].

The CIDT has already been validated and found effective for identifying people with potential mental health problems and promoting help-seeking behavior in Nepal [19, 22]. After the 2015 Nepal earthquakes, the CIDT was adapted for two additional conditions, suicide and post-traumatic stress, and was used in five earthquake affected districts covering a population of approximately 1 million [22]. We found that the CIDT approach could be rapidly adapted for other conditions. To employ CIDT in settings outside Nepal, we recommend a similar adaptation procedure to the one outlined here with regards to importance of identifying local idioms and piloting vignettes. Moreover, the ideal cadres are likely to vary from setting to setting. In Nepal, FCHVs had the most community contact. In other settings, this may be social workers, community mobilizers, or other health worker cadres. Therefore, contextualization should take into account who would be best positioned to employ this tool. It would be noteworthy if future researches focus on testing the feasibility of the tool in terms of cost and time requirements. A final important consideration is that CIDT appeared to work well in part because of lack of preexisting mental health literacy in the community and widespread availability of a human resource with strong community connections. In settings lacking such conditions, other approaches to facilitating treatment engagement may be more appropriate.

## Conclusion

A context specific and culturally appropriate tool was developed by following a systematic approach to facilitate detection of five mental health problems in the community. Although CIDT is not an alternative to standard screening tools, it can play an important role in promoting help-seeking behaviors among people with potential mental health problems through early detection and referral at the community level. The tool is simple and can be used by anyone with limited literacy skills provided a brief training prior to its implementation is conducted. Considering the current workload of FCHVs as volunteers, provision of small incentives can increase their motivation to use the CIDT. Except for behavioral disorders in children, CIDT can be used by community informants to identify potential cases of the mental health problems targeted. Mobilizing children’s family member or primary caregiver is recommended for case detection of child behavioral problems.

## Additional file

Additional file 1: Community Informant Detection Tool (CIDT) for Depression. (DOCX 597 kb)

## Abbreviations

CIDT: Community Informant Detection Tool
FCHV: Female Community Health Volunteers
IDI: In-depth interview
LMIC: Low and Middle Income Countries
mhGAP: Mental health Gap Action Programme
PRIME: Programme for Improving Mental Health Care
VDC: Village Development Committee
WHO: World Health Organization
