# Supplementary material for: Improving detection of mental health problems in community settings in Nepal: development and pilot testing of the community informant detection tool
Source: Confl Health. 2017 Nov 20;11:28. doi: 10.1186/s13031-017-0132-y (PMC5694900; doi:10.1186/s13031-017-0132-y)
Supplement: Additional file 1: — Community Informant Detection Tool (CIDT) for Depression. (DOCX 597 kb) [file 13031_2017_132_MOESM1_ESM.docx]

**Additional File 1– Community Informant Detection Tool (CIDT) for Depression** ****
